# Treatment delays in oncology patients during COVID-19 pandemic: A perspective

**DOI:** 10.7189/jogh.10.010367

**Published:** 2020-06

**Authors:** Divyesh Kumar, Treshita Dey

**Affiliations:** Department of Radiotherapy and Oncology, PGIMER, Chandigarh, India

Ever since the emergence of novel coronavirus disease 2019 (COVID-19) in Wuhan, China, it has impacted mankind globally. WHO declared it a public health emergency since March 11, 2020 [1]. A major effect of it has been seen in the health sector worldwide, due to the unpreparedness for such an event. Lockdown and social distancing, though necessary non-pharmacological measures to flatten the transmission curve, have further aggravated the interruption of health care services to the needy. Worse survival has been reported in oncology patients during the COVID crisis, due to their compromised immunity, which could be disease or anticancer treatment-related. As a considerable fraction of patients visiting the health care institute are oncology patients, interruption of health care services during the crisis might delay treatment in these patients.

Delays during oncological treatment have been classified as primary, secondary, and tertiary [2]. A significant proportion of time is usually consumed in primary (interval between symptom onset to the first visit to the clinician) and secondary delay (interval between clinician visit to start of treatment) in a majority of oncology patients awaiting treatment, especially in low-middle income countries (LMIC) with huge patient burden. Since time factor is an important element in oncology treatment, delivering optimal care during the COVID-19 pandemic is a challenging event. As oncology care hangs on a fine-scale balance amidst the COVID pandemic, striking a balance between delivering or delaying treatment during this crisis, becomes crucial not only for oncology patients but also for the treating clinician. Various factors contributing to treatment delays, its possible impact on the oncology patients and clinicians’ role during the COVID-19 crisis, is henceforth worth discussing and needs to be highlighted.

## FACTORS DELAYING TREATMENT

Factors that might delay treatment can be grouped into two categories (**Box 1**).

Box 1 **Factors delaying treatment**

Patient-related factors:

• Travel inconvenience due to lockdown

• Financial issues

• Patients coming from distant places for treatment

• Accommodation and food-related issues

Healthcare-related factors:

• Delays in surgery

• Shortage of personal protective equipment (PPE) & ventilators

• Manpower shortage

### Patient-related factors

Social containment and travel restrictions during the pandemic, imposes difficulties for patients to attend the hospital for treatment. Also, the financial issues faced by poor strata are a big challenge amongst cancer patients, especially in LMIC. Furthermore, some patients come from far off place for treatment, propelling them to look for accommodation and food facilities. Amidst the lockdown, arranging for food and shelter is another biggest hurdle faced by these patients. Allocation of in-patient facilities to the COVID patients, due to the huge case burden, further aggravates this situation.

### Healthcare-related factors

With the increase in surge of COVID-19 patients, hospitals are forced to allocate resources and oncology surgeries to be delayed. Of late, due to the rising trend of this pandemic there has been an acute shortage of ventilators as well as PPE, causing further surgical delays. Also, a shortage of staff members dealing with oncology treatment execution leads to unwanted delays.

## ADVANTAGES OF TREATMENT DELAYS DURING CRISIS

Some positive effects of treatment delays during the crisis phase could be:

(i) Vulnerable patients, ie, elderlies and those with comorbidities, can be kept out of hospitals and treatment facilities, as they are prone to get infected.

(ii) Immune-dampening effects of radiotherapy (RT) and/or systemic therapy can be curtailed.

## IMPACT ON ONCOLOGY PATIENTS

These can be grouped into three categories (**Box 2**).

Box 2 **Impact of treatment delay according to treatment schedule.**

Impact of delay in treatment-naive patients:

• Adversely affects survival and quality of life

• Psychological impact

Impact of delay in patients on treatment:

• Accelerated repopulation

• Resistance to treatment

Impact of delay on follow-up (FU) patients:

• Delay in detecting treatment response

• Delay in the diagnosis of recurrence

### Impact of delay in treatment-naive patients

(i) **Impact of delay in definitive treatment:** Surgery, RT, and chemotherapy (CT) either alone or in combination forms the definitive modality of treatment for most of the malignancies. Delay in the initiation of radical treatment might lead to a decrease in locoregional control and overall survival (OS). Chen et al. in their study inferred that an increase in waiting time for RT is generally associated with deterioration in local control rates and OS [3]. Similarly, an adverse impact on survival has been documented in patients with delays in CT and surgery [4,5]. Hence, extending the treatment time of definitive treatment can have detrimental effects on the expected treatment response and quality of life (QOL) of the oncology patients.

(ii) **Psychological impact:** Fear of disease progression or recurrence is quite common amongst patients awaiting oncology treatment. Elevated levels of fear of progression can affect patients’ well-being, QOL, and social functioning [6]. Treatment delays, thus, can lead to psychological stress, hampering their QOL.

### Impact of delay in patients on treatment

RT and CT are usually given at intervals to allow sufficient time for normal cells to undergo repair of the sub-lethal damage. Surviving tumor cells have the propensity to proliferate during treatment breaks. Chances of accelerated repopulation of tumor cells increases in patients with delays in ongoing CT/RT [7,8]. Patients are thus prone to increased chances of disease recurrence and treatment resistance.

Also, patients with terminal illness requiring hospice admission and strong opioids during palliative care might land up with aggravations of symptoms, in case of delays in palliative care treatment, if any. Treatment interruptions, thus, can lead to progressive symptoms and worsened survival chances.

### Impact of delay on FU patients

FU is an integral part of the management of oncology patients. It is essential for assessing clinical response, late effects of treatment, detecting residual/recurrences, second malignancy, and symptomatic and supportive treatment if required [9]. Treatment delay, thus refrain them from the mentioned advantages of FU. Mostly affected in this group during the pandemic, are the patients who develop symptoms of recurrences or second malignancy, requiring further diagnostic and treatment interventions.

**Figure:**
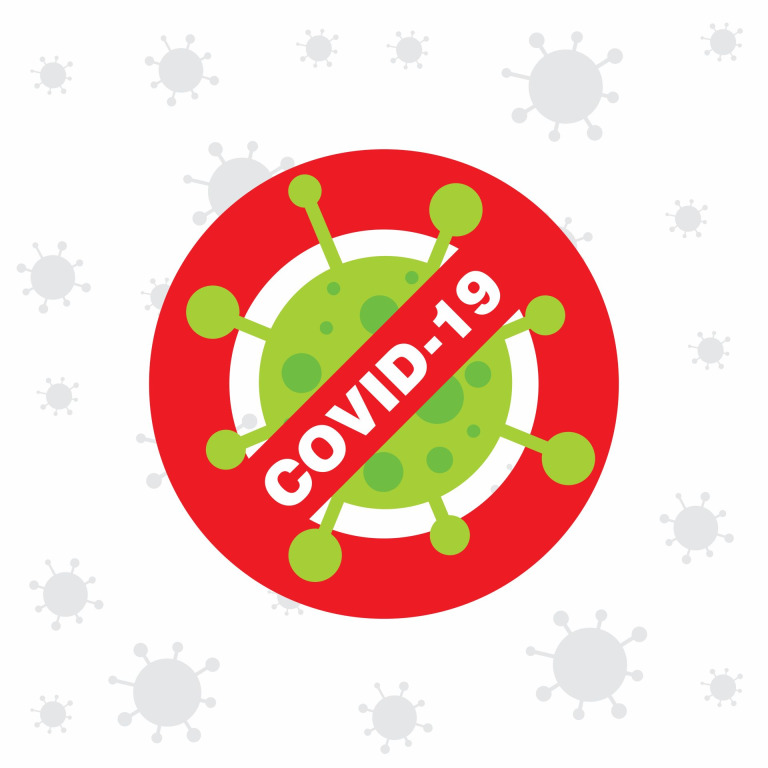
Photo: Stop vector created by flatart (source: https://www.freepik.com/free-photos-vectors/stop).

## CLINICIAN’S ROLE DURING THE CRISIS

The oncology clinicians have a very important role to play during the crisis (**Box 3**).

Box 3 **Clinician’s role during crisis**

Identifying tumor type and risk stratification approach:

• Appropriate rationalized treatment

Counseling:

• Improve clinician-patient relation

Amendments in treatment protocols:

• Altered fractionation

Telemedicine approach:

• Especially for follow-up

### Identifying tumor type and risk stratification approach

As different tumor histologies vary in their reproductive capability, it is the rapidly proliferating tumor cells that are of utmost concern. Hence, a risk stratification approach according to tumor subtype can be followed. In a study published by Kutikov. A et al., a risk-based conceptual approach regarding oncology treatment during the COVID-19 pandemic has been highlighted [10].

### Counseling

An important way to reduce the mental stress amongst the oncology patients is by a good clinician-patient relationship. An effective counseling is thus, of immense importance for these patients. Mostly, patients diagnosed with benign conditions, early-stage or low-grade malignancy, and post-surgery patients requiring only FU are usually benefitted the most.

### Amendments in treatment protocols

To reduce overall treatment time to shorten hospital visits, altered fractionation regimen, especially hypofractionated or accelerated radiotherapy regimens, wherever feasible, may be followed.

### Telemedicine approach

Telemedicine has emerged as a boon amidst this crisis. All queries of patients and attendants could be adequately addressed through a telemedicine approach. Especially, those patients on regular FU, may be managed by this approach and made to defer unnecessary visits to hospitals. Only if, deemed necessary they should be asked to visit the hospital during the crisis.

## CONCLUSIONS

Although factors causing treatment delays, might have a negative impact on the survival and QOL in oncology patients, advantages associated with it makes it paramount to carefully weigh the risk of treatment delays vs benefits of continuing therapy during the crisis. A detailed discussion and counseling of patients regarding the possible outcomes of treatment interruption on disease control and its effect on survival might help in overcoming the associated stress. Optimal patient care should be targeted by identifying tumor type and appropriate risk stratification approach. Furthermore, all possible measures to curtail the crisis such as triaging to identify the critical cases, amendments in treatment protocols, and telemedicine facilities should be sought.
